# Increased Monocyte Inflammatory Responses to Oxidized LDL Are Associated with Insulin Resistance in HIV-Infected Individuals on Suppressive Antiretroviral Therapy

**DOI:** 10.3390/v12101129

**Published:** 2020-10-05

**Authors:** Brooks I. Mitchell, Elizabeth I. Laws, Dominic C. Chow, Ivo N. Sah Bandar, Louie Mar A. Gangcuangco, Cecilia M. Shikuma, Lishomwa C. Ndhlovu

**Affiliations:** Hawaii Center for AIDS, John A. Burns School of Medicine, University of Hawai’i, Honolulu, HI 96813, USA; brooksim@hawaii.edu (B.I.M.); lawse@hawaii.edu (E.I.L.); dominicc@hawaii.edu (D.C.C.); ins4002@med.cornell.edu (I.N.S.B.); louiemag@hawaii.edu (L.M.A.G.); shikuma@hawaii.edu (C.M.S.)

**Keywords:** insulin resistance, HIV, monocytes, inflammation

## Abstract

Despite long term antiretroviral therapy (ART), insulin resistance (IR) is common among people living with HIV/AIDS (PLWHA) exposing this population to a greater risk of cardiometabolic complications when compared to their uninfected counterparts. We previously identified an expansion in monocyte subpopulations in blood that were linked to the degree of IR in persons with HIV on stable ART. In this study, we directly assessed monocyte inflammatory functional properties from PLWHA on ART (*n* = 33) and HIV-uninfected controls (*n* = 14) of similar age, gender, and cardiovascular disease risk and determined the relationship with IR (homeostatic model assessment-insulin resistance (HOMA-IR)), calculated from fasting blood glucose and insulin measurements. Peripheral blood mononuclear cells were stimulated with oxidized low-density lipoproteins (oxLDL) and polyfunctional monocyte cytokine responses (IL-1β, IL-6, IL-8, or TNF-α) were determined by flow cytometry. Higher monocyte IL-1β and IL-8 responses to oxLDL were associated with higher IR in PLWHA but not in the control group. We observed that higher basal monocyte cytokine responses were associated with both duration since HIV diagnosis and ART initiation. In the management of IR in chronic HIV, strategies lowering monocyte IL-1β and IL-8 responses should be considered in addition to ART in order to limit adverse cardio-metabolic outcomes.

## 1. Introduction

With the advances of combination antiretroviral therapy (ART), HIV infection is no longer defined by AIDS-related illnesses and short life expectancy, but now defined as a chronic disease of emerging cardio-metabolic complications [1]. People living with HIV/AIDS (PLWHA) on long term suppressive ART have increased rates of metabolic abnormalities, such as insulin resistance (IR) and type 2 diabetes mellitus (T2DM) [2], which have been associated with higher risk of cardiovascular disease (CVD) [3,4,5]. The etiologies of IR and T2DM are multifactorial in PLWHA and includes the effects of lipodystrophy, co-infection with hepatitis C virus, mitochondrial dysfunction, and ART; in addition to increased rates of traditional risk factors, such as obesity [1,2,6,7].

Inflammation and immune dysfunction driven by chronic HIV infection during suppressive ART may also play a role in the prevalence of these metabolic conditions [8]. C-reactive protein (CRP) and soluble receptors of tumor necrosis factor-alpha (TNF-α), sTNF1, and sTNF2, are found to be associated with the development of T2DM in PLWHA after ART initiation [9]. We have shown in a cross-sectional analysis of 141 PLWHA on suppressive ART enrolled in the Hawaii Aging with HIV-Cardiovascular (HAHC-CVD) cohort that plasma levels of tissue plasminogen activator inhibitor-1 (tPAI-1) was elevated among participants with higher homeostatic model assessment of insulin resistance (HOMA-IR) [10]. Furthermore, soluble E-selectin (sE-selectin) and soluble intercellular cell adhesion molecule-1 (sICAM-1) were found to be elevated among participants with metabolic syndrome; and plasma myeloperoxidase (MPO), monocyte chemoattractant protein-1 (MCP-1), and vascular endothelial growth factor (VEGF) were elevated among participants with T2DM relative to those with normal glucose tolerance [10].

Monocytes have been implicated in HIV pathogenesis [11,12] and in the development of non-AIDS related comorbidities, including atherosclerosis [13,14,15] and cognitive impairment [16,17,18,19,20]. We have previously shown that an increase in total blood monocytes is associated with elevated homeostatic model assessment of insulin resistance (HOMA-IR) in PLWHA on stable ART, independent of HIV immuno-virologic and traditional T2DM risk factors [10]. We have also shown that PLWHA on ART regimens have higher percentages of monocytes producing intracellular IL-1β and IL-8 in both the basal state and upon stimulation as compared to HIV-uninfected participants similar in age, gender, and CVD risk [21]. More recently we observed a distinct methylomic signature in peripheral monocytes that is associated with insulin resistance in PLWHA. The 123 CpGs identified in monocytes that were significantly different in DNA methylation, were enriched at genes relating to glucose metabolism, immune activation, and insulin signaling. Furthermore, using logistic regression models, methylation levels of 4 monocyte-derived CpGs were found to independently predict an IR state. Together, these results support a strong link between monocytes and IR.

Elucidation of the mechanisms underlying monocyte changes contributing to the development of IR and T2DM in PLWHA on suppressive ART is an approach to better understand monocyte immunopathology. Here, we explored the relationship between monocyte cytokine responses to HIV-associated immunologic and cardio-metabolic parameters in PLWHA on stable ART and HIV-uninfected controls similar in age, gender, race, and BMI.

## 2. Materials and Methods

### 2.1. Study Participants and Study Design

Thirty-three PLWHA and 14 HIV-uninfected participants (controls) were studied from the Hawaii Aging with HIV Cohort-Cardiovascular Disease (HAHC-CVD) study [21,22] with available monocyte cytokine responses and analyzed in reference to additional available clinical, HIV immuno-virologic and cardio-metabolic data from these participants (Table 1). PLWHA who entered into the HAHC-CVD study were ≥40 years old and were on stable ART ≥ 3 months. The 14 controls were similar in age and gender and underwent similar clinical and immunological assessments as the PLWHA group. HOMA-IR was calculated [23], and participants were characterized as having T2DM using the American Diabetic Association Guidelines [24]. Metabolic syndrome in HAHC-CVD was defined using the criteria proposed by the National Cholesterol Education Program’s Adult Treatment Panel III report (ATP III) [25]. IRB approval was obtained from the University of Hawaii Human Studies Program.

### 2.2. Assessment of Monocyte Intracellular Cytokine Production

Monocyte cytokine responses at basal level and following oxLDL stimulation were evaluated by multi-parametric flow cytometry as previously described [21].

### 2.3. Statistical Analysis

Comparisons between PLWHA and controls were calculated using Wilcoxon rank and chi-square tests for continuous and categorical variables, respectively. Pearson product-moment correlation and multivariable linear regression were utilized to assess associations. A two-sided probability of *p*-value < 0.05 was considered statistically significant. Statistical analyses were performed using the SPSS statistical program (SPSS Statistics 23, Armonk, NY, USA).

## 3. Results

The characteristics of the 33 PLWHA and 14 controls are shown in Table 1. Of the PLWHA on ART, the majority were virally suppressed. HOMA-IR and the number of participants with T2DM and metabolic syndrome were only slightly higher in the PLWHA group and there were no statistical differences observed when compared to the control group.

We assessed in PLWHA the relationships of CD4 and CD8 T cell parameters to percentages of monocyte cytokine responses at both basal levels and after oxLDL stimulation (Table 2). Nadir CD4 T cell counts showed no correlations with percentages of IL-1β+, IL-8+, IL-6+, and TNF-α+ monocytes in either condition. While absolute current CD4 T cell counts did not correlate to monocyte responses, current CD4 T cell percentages negatively correlated with basal percentages of IL-6+ monocytes (*r* = −0.389, *p* = 0.025) and oxLDL-stimulated percentages of IL-8+ and TNF-α+ monocytes (IL-8; *r* = −0.523, *p* = 0.002 and TNF-α; *r* = −0.410, *p* = 0.018). No correlations were seen with current CD8 T cell percentages; however absolute current CD8 T cell counts correlated positively with basal percentages of IL-6+ monocytes (*r* = 0.368, *p* = 0.035) and oxLDL-stimulated percentages of IL-8+ monocytes (*r* = 0.460, *p* = 0.007). Activated CD8 T cell counts correlated only with basal percentages of IL-6+ monocytes (*r* = 0.513, *p* = 0.002), while activated CD8 T cell percentages showed no correlations with monocyte cytokine responses in either condition. CD4/CD8 T cell count ratios correlated negatively with IL-8 (*r* = −0.501, *p* = 0.003) and TNF-α (*r* = −0.417, *p* = 0.016) monocyte responses after oxLDL stimulation.

Interestingly, duration since HIV diagnosis correlated positively with basal percentages of IL-1β+ (*r* = 0.345, *p* =0.049), IL-6+ (*r* = 0.410, *p* = 0.018), and TNF-α+ (*r* = 0.403, *p* = 0.020) monocytes (Table 2). Duration since ART initiation correlated positively with basal percentages of IL-6+ (*r* = 0.503, *p* = 0.003) monocytes. None of the monocyte cytokine responses in stimulated conditions correlated with duration since HIV diagnosis or with duration since ART initiation.

Basal and oxLDL-stimulated monocyte responses showed no correlation with total cholesterol, HDL, and LDL cholesterol levels in either PLWHA or control groups. Triglyceride levels correlated only with oxLDL-stimulated percentages of IL-1β+ and IL-8+ monocytes in PLWHA (IL-1β; *r* = 0.489, *p* = 0.004 and IL-8; *r* = 0.414, *p* = 0.017) (Table 2). In the control group, no correlations were observed between triglyceride levels and monocyte cytokine responses in either basal or stimulated conditions.

PLWHA with metabolic syndrome had higher percentages of IL-1β+ monocytes after oxLDL stimulation (*p* = 0.018) as compared to PLWHA without metabolic syndrome. No differences in percentages of IL-6+, IL-8+, or TNF-α+ monocytes after oxLDL stimulation or basal monocyte responses were observed. No differences were seen in the control group.

**Figure 1:**
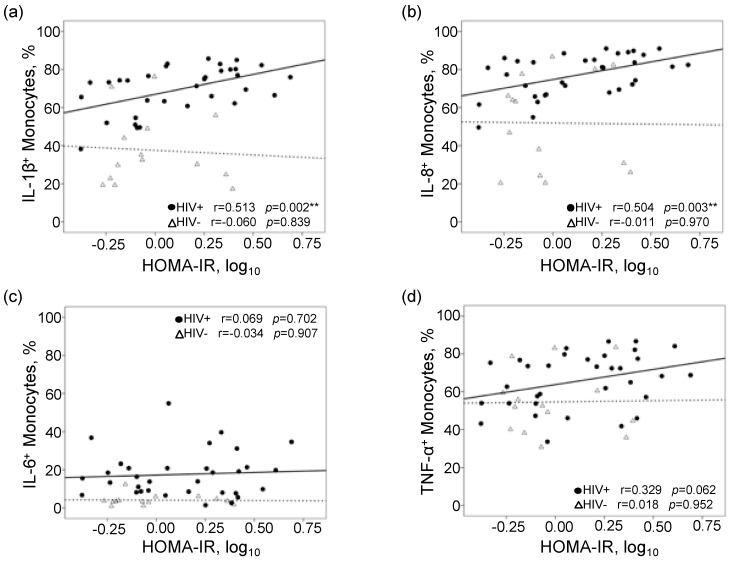
Scatterplots and Pearson correlations of frequencies of intracellular cytokine-producing monocytes to homeostatic model assessment-insulin resistance (HOMA-IR). Correlation plots of frequencies of (**a**) IL-1β+, (**b**) IL-8+, (**c**) IL-6+, and (**d**) TNF-α+ monocytes (oxidized LDL stimulated) to HOMA-IR. Solid line represents best fit line for PLWHA (*n* = 33) and dashed line represents best fit line for controls (*n* = 14). ** *p* < 0.01.

There were no correlations between HOMA-IR and basal monocyte responses in either the PLWHA or control groups. However, when stimulated monocyte responses were analyzed, we observed strong positive correlations between HOMA-IR and percentages of IL-1β+ (*r* = 0.513, *p* = 0.002) and IL-8+ (*r* = 0.504, *p* = 0.003) monocytes among PLWHA (Figure 1a,b). The strong correlations continued to be seen when the four participants with T2DM were excluded. Furthermore, the significant correlations remained when the five participants with detectable plasma HIV RNA were excluded. In contrast, no associations were seen between HOMA-IR and IL-1β+ (*r* = 0.060, *p* = 0.837) and IL-8+ (*r* = 0.011, *p* = 0.891) monocytes in the control group. A trend towards a significant correlation was seen between percentages of TNF-α+ monocytes and HOMA-IR only in PLWHA (*r* = 0.329, *p* = 0.074), while no correlations were seen for percentages of IL-6+ monocytes in either group (Figure 1c,d).

We attempted to assess the independent predictive value of percentages of IL-1β+ and IL-8+ monocytes in explaining increased HOMA-IR when controlled for other variables associated with IR in this population. Because of the small number in our PLWHA group, it was not statistically possible to control for all parameters of interest. In univariable linear regression analyses, a significant predictive value was seen only for BMI (β = 2.028, *p* = 0.005, CI = 0.642–3.414) with no significant predictive value seen for other parameters associated with the development of IR in HIV: age, gender, ethnicity (Caucasian vs. non-Caucasian), current or nadir CD4 T cell count, HIV RNA, Hepatitis C infection, duration of HIV infection, or history of protease inhibitor or nucleoside reverse transcriptase inhibitor zidovudine or stavudine use. In multivariable linear regression models controlled for BMI, percentages of IL-1β+ and IL-8+ monocytes in PLWHA continued to significantly predict higher HOMA-IR (IL-1β; β = 1.875, *p* < 0.0005, CI = 0.940–2.810 and IL-8; β = 1.915, *p* = 0.007, CI = 0.567–3.264). Inclusion of age, ethnicity, and current or nadir CD4 T cell counts into the models gave similar results.

## 4. Discussion

Our present study suggests a novel association between stimulated IL-1β and IL-8 monocyte responses in peripheral blood and increased IR among PLWHA on stable ART regimens. These associations were found to be independent of BMI and were not present among the control group. Interestingly, we also found un-stimulated monocyte responses to be higher in individuals with longer durations since HIV diagnosis or since ART initiation.

IL-1β is one of the major pro-inflammatory cytokines produced by monocytes/macrophages and is shown to be an important mediator in a number of acute and chronic inflammatory diseases including T2DM in the general population [26]. After prolonged exposure to IL-1β in vitro, induced insulin resistance has been reported in murine and human adipocytes through a decrease in the expression of the glucose transporter Glut 4, which ultimately impairs insulin signaling and action [27]. Others report a cytotoxic effect of IL-1β on insulin-producing pancreatic β cells [26]. Overall, our findings implicate a role for monocyte derived IL-1β as a potential mechanism in the increased risk for IR and metabolic disease among PLWHA.

As with IL-1β, IL-8 is a pro-inflammatory cytokine produced by a number of cell types, including monocytes/macrophages, and has been reported to inhibit Akt phosphorylation, increasing IR in human adipocytes [28]. While we did not observe a significant relationship between monocyte IL-6 and TNF-α responses with IR, IL-6 and TNF-α have been reported to be involved in the development of IR [29,30,31] and have a synergistic relationship with IL-1β and IL-8 responses, further driving IR [21,27,28,29,31,32].

Targeted treatments towards IL-1β in humans have been described for T2DM and other inflammatory disease states in the general population [26]. A neutralizing IL-1β antibody was well tolerated by T2DM participants and showed modest reduction of HbA1c, fasting glucose, and inflammatory markers [33]. Similarly, Gevokizumab, a human-engineered IL-1β monoclonal antibody, showed improvement in glycemia and reduction of inflammation in T2DM patients [34]. Reductions in HbA1c, systemic inflammatory markers, as well as proinsulin to insulin ratios in T2DM patients, were demonstrated after 13 weeks of Anakinra administration, a recombinant IL-1R antagonist [35]. Given our study findings, the effect of IL-1β targeted therapeutics on monocyte cytokine responses in HIV may be of interest. Our previous report revealed a positive relationship between monocyte IL-1β and IL-8 responses [21], thus targeting IL-8 as a downstream mediator of inflammation [36,37,38,39] in conjunction with targeting IL-1β may also be considered.

Our study found an expected correlation between immune dysregulation as measured by CD4/CD8 ratio [40] and monocyte cytokine responses. Unexpectedly, we found strong correlations (explaining 9 to 25% of the variance) between monocyte cytokine responses and either duration since HIV diagnosis or since ART initiation independent of CD4/CD8 ratio. Both duration of disease/ART as well as the extent of HIV immune dysregulation may be important in explaining the degree of monocyte cytokine responses. Longitudinal studies may be warranted to examine the validity of this association. The increase in monocyte cytokine responses with time may be reflective of increased inflammation associated with elevated cellular turnover and senescence of monocytes [41]. We were not able to decouple the duration effects due to the close relationship between the two parameters, both of which were by self-report. We did not detect a direct relationship between both durations to HOMA-IR, but this may be a limitation of our sample size.

This study is limited by its relatively small sample size and conclusions made between PLWHA and control groups require to be further examined in studies with larger sample groups. However, the strengths of the study are the careful clinical and cardio-metabolic characterizations performed on the PLWHA and control groups, as well as detailed intracellular cytokine phenotyping of monocytes that reveal discriminating associations in PLWHA.

In conclusion, higher stimulated monocyte cytokine responses, particularly in IL-1β and IL-8, are associated with increases in IR in PLWHA but not in uninfected controls. Duration since HIV diagnosis or since ART initiation may contribute to dysregulated monocyte cytokine responses. In the management of IR in chronic HIV, strategies lowering monocyte IL-1β and IL-8 responses should be considered in addition to ART in order to limit adverse cardio-metabolic outcomes.

## Figures and Tables

**Table 1:** Comparison of baseline measures of study participants ^1^.

Baseline Measures	PLWHA *n* = 33	Controls *n* = 14	*p*-Value
Age, years	53 (49, 56)	51 (46, 60)	0.552
Male, *n* (%)	29 (88%)	14 (100%)	0.302
Caucasian, *n* (%)	22 (67%)	9 (64%)	1.000
Body mass index, kg/m^2^	26 (23, 27)	24 (23, 27)	0.601
History of smoking, *n* (%)	22 (67%)	11 (79%)	0.724
History of hypertension, *n* (%)	10 (30%)	4 (29%)	0.516
HOMA-IR	1.46 (0.79, 2.48)	0.85 (0.62, 1.74)	0.129
Metabolic syndrome, *n* (%)	7 (21%)	1 (7%)	0.405
Type 2 Diabetes Mellitus, *n* (%)	4 (12%)	0	0.302
Total cholesterol, mg/dL	175 (146, 189)	173 (151, 192)	0.658
HDL cholesterol, mg/dL	36 (30, 45)	55 (46, 64)	0.001
LDL cholesterol, mg/dL	101 (81, 122)	107 (86, 114)	0.585
Triglycerides, mg/dL	125 (83, 161)	78 (56, 140)	0.076
Hepatitis C infection, *n* (%)	5 (15%)	0	0.303
Nadir CD4^+^ T cells, cells/μL	181 (63, 275)	-	-
CD4^+^ T cells, cells/μL	574 (450, 713)	-	-
CD4^+^ T cells, %	33 (24, 37)	-	-
CD8^+^ T cells, cells/μL	801 (594, 1087)	-	-
CD8^+^ T cells, %	43 (35, 50)	-	-
Activated CD8^+^ T cells (CD38^+^HLA-DR^+^), cells/μL	83 (56, 161)	-	-
Activated CD8^+^ T cells (CD38^+^HLA-DR^+^), %	12 (9, 17)	-	-
HIV RNA < 50 copies/mL, *n* (%)	28 (85%)	-	-
Duration since HIV diagnosis, years	16 (8, 23)	-	-
Duration since ART initiation, years	12 (6, 15)	-	-
History of NRTI use, *n* (%)	33 (100%)	-	-
History of NNRTI use, *n* (%)	23 (70%)	-	-
History of Protease Inhibitor use, *n* (%)	21 (64%)	-	-

^1^ Values are shown in median (interquartile range) or frequency, *n* (%). People living with HIV/AIDS (PLWHA); homeostatic model assessment of insulin resistance (HOMA-IR); high-density lipoprotein (HDL); low-density lipoprotein (LDL); cluster of differentiation (CD); human leukocyte antigen-DR isotype (HLA-DR); human immunodeficiency virus (HIV); ribonucleic acid (RNA); antiretroviral therapy (ART); nucleoside/nucleotide reverse transcriptase inhibitor (NRTI); non-nucleoside reverse transcriptase inhibitor (NNRTI).

**Table 2:** Correlations between monocyte inflammatory responses and clinical parameters in people living with HIV/AIDS (PLWHA).

Variable	Unstimulated (Basal) Monocyte Response				Oxidized LDL-Stimulated Monocyte Response			
	IL-1β+	IL-8+	IL-6+	TNF-α+	IL-1β+	IL-8+	IL-6+	TNF-α+
Total cholesterol	*r* = −0.238	*r*= −0.017	*r*= 0.180	*r* = −0.344	*r* = −0.240	*r* = 0.017	*r* = −0.174	*r* = 0.167
HDL cholesterol	*r* = 0.233	*r* = 0.125	*r* = −0.024	*r* = −0.167	*r* = −0.284	*r* = −0.223	*r* = −0.002	*r* = 0.042
LDL cholesterol	*r* = −0.227	*r* = −0.102	*r* = 0.145	*r* = −0.166	*r* = −0.270	*r* = 0.013	*r* = −0.280	*r* = 0.197
Triglycerides	*r* = −0.168	*r* = 0.060	*r* = 0.232	*r* = −0.167	*r* = 0.489 *	*r* = 0.414 *	*r* = 0.235	*r* = −0.152
Nadir CD4 T cells	*r* = −0.027	*r* = −0.037	*r* = −0.291	*r* = −0.196	*r* = 0.209	*r* = 0.021	*r* = 0.240	*r* = 0.033
CD4^+^ T cells	*r* = 0.279	*r* = 0.241	*r* = 0.013	*r* = 0.227	*r* = 0.100	*r* = −0.054	*r* = 0.267	*r* = −0.181
CD4^+^ T cells (%)	*r* = 0.062	*r* = −0.095	*r* = −0.389 *	*r* = 0.142	*r* = −0.223	*r* = −0.523 *	*r* = 0.069	*r* = −0.410 *
CD8^+^ T cells	*r* = 0.191	*r* = 0.241	*r* = 0.368 *	*r* = 0.054	*r* = 0.297	*r* = 0.460 *	*r* = 0.245	*r* = 0.142
CD8^*^ T cells (%)	*r* = −0.032	*r* = 0.040	*r* = 0.244	*r* = 0.114	*r* = −0.041	*r* = 0.261	*r* = −0.021	*r* = 0.150
CD4/CD8 T cell ratio	*r* = 0.079	*r* = −0.011	*r* = −0.295	*r* = 0.057	*r* = −0.180	*r* = −0.501 *	*r* = 0.146	*r* = −0.417 *
Activated CD8^+^ T cells	*r* = 0.224	*r* = 0.292	*r* = 0.513 *	*r* = 0.026	*r* = 0.113	*r* = 0.190	*r* = 0.266	*r* = −0.004
Activated CD8^+^ T cells (%)	*r* = 0.072	*r* = 0.051	*r* = 0.211	*r* = 0.080	*r* = −0.075	*r* = −0.130	*r* = 0.103	*r* = −0.188
Duration since HIV diagnosis	*r* = 0.345 *	*r* = 0.161	*r* = 0.410 *	*r* = 0.403 *	*r* = 0.092	*r* = 0.181	*r* = −0.132	*r* = −0.238
Duration since ART initiation	*r* = 0.178	*r* = 0.229	*r* = 0.503 *	*r* = 0.133	*r* = 0.136	*r* = 0.311	*r* = −0.004	*r* = −0.043

* *p*-value < 0.05. Interleukin (IL); tumor necrosis factor (TNF).
